# Inguinal Intranodal Lymphangioma in an Adult: A Clinical Case Report

**DOI:** 10.7759/cureus.50402

**Published:** 2023-12-12

**Authors:** Alejandra C Vásquez-Castillo, Justin Moreira, Jesus E Guarecuco Castillo, Feisal Hamam, Mohammed M Masri

**Affiliations:** 1 Osteopathic Medicine, Lake Erie College of Osteopathic Medicine, Bradenton, USA; 2 General Surgery, Ross University School of Medicine, Christ Church, BRB; 3 Surgery, Larkin Community Hospital, Florida, USA; 4 Surgery, Larkin Community Hospital, South Miami, USA; 5 General Surgery, Larkin Community Hospital, South Miami, USA

**Keywords:** inguinal intranodal lymphangioma, inguinal lymphangioma, lymphatic malformation, adult lymphangioma, surgical case reports

## Abstract

Lymphangiomas are benign deformities of the lymphatic system that are most common in pediatric populations and are usually found in the neck, axilla, chest wall, cervicofacial, and pelvic regions, as they are areas with more lymphatic activity. Herein, we report the case of a 43-year-old African-American male who presented with bilateral inguinal lymphangiomas, the first documented incidence of its kind. This patient presented with several months of bilateral swellings in his groin area, accompanied by increased tenderness and discomfort. A physical exam revealed that the left groin swelling was larger than the right groin swelling and that the left lymph node was about < 2 cm in size. Bilaterally, there was no tenderness of the lymph nodes in the area and no skin changes, ulceration, induration, discharge, or bleeding from the site. The diagnostic assessment included ultrasound, a left inguinal lymphadenectomy, and a frozen section biopsy to provide a definitive diagnosis. The pathology report described the lesion as a benign lymphangioma and was negative for lymphoproliferative lesions.

## Introduction

Lymphangiomas are non-malignant malformations of the lymphatic system that can present on the skin, mucous membranes, or extranodal locations. Although they are most often present in pediatric patients as congenital malformations, they can rarely be acquired [1]. Most commonly, these deformities do not require invasive treatment, but if functional compromise or complications due to mass effect develop, surgical excision is the treatment of choice [1,2]. We present a case where bilateral intranodal inguinal lymphangiomas were detected in an adult, an atypical presentation not previously documented in the literature. They were addressed with a left-sided resection. This case emphasizes the importance of considering lymphangiomas in adult patients who present with masses in lymphodense areas.

## Case presentation

The patient is a 43-year-old African-American male referred for general surgery consultation by his primary care physician for bilateral groin swelling. He had been noticing this swelling for more than six months and sought an evaluation after experiencing increased tenderness and overall discomfort in this area. His only known medical history includes a herpes simplex virus infection for which he took an unspecified medication and a surgical history of left total orchidectomy after suffering from testicular torsion. Additionally, the patient was taking amlodipine besylate oral tablet 10 mg by mouth daily and had no known allergies. This patient's family history was non-contributory.

Since the onset of the swelling, the patient did not report an increase in swelling, but concerns for increased tenderness and overall discomfort led to his evaluation. A full review of the systems only revealed abnormal blood pressure, shortness of breath, and a productive cough. Furthermore, the patient was negative for penile discharge, scrotum swelling, or scrotal mass presence. When the patient arrived, he was alert and oriented to time, place, and person. He appeared consistent with his age and well-nourished. During this time, the patient had no fever, sweats, chills, fatigue, or unintentional weight loss. Upon physical examination, it was noted that the swelling in the left groin area was more prominent, with lymph nodes measuring less than 2 cm upon deep palpation when compared to the right. The lymph nodes were non-tender to palpation. There were no skin changes, ulceration, discharge, or bleeding from the site, and no induration was present. During the physical exam, it was also noted that the patient had an absent left testicle and a visible scar over the left scrotum. The remainder of the physical exam was unremarkable. Although differential diagnoses of tuberculosis, sexually transmitted diseases (STDs), and malignancy were considered, the patient's denial of fever, weight loss, and chronic cough made these diagnoses less likely.

**Figure 1:**
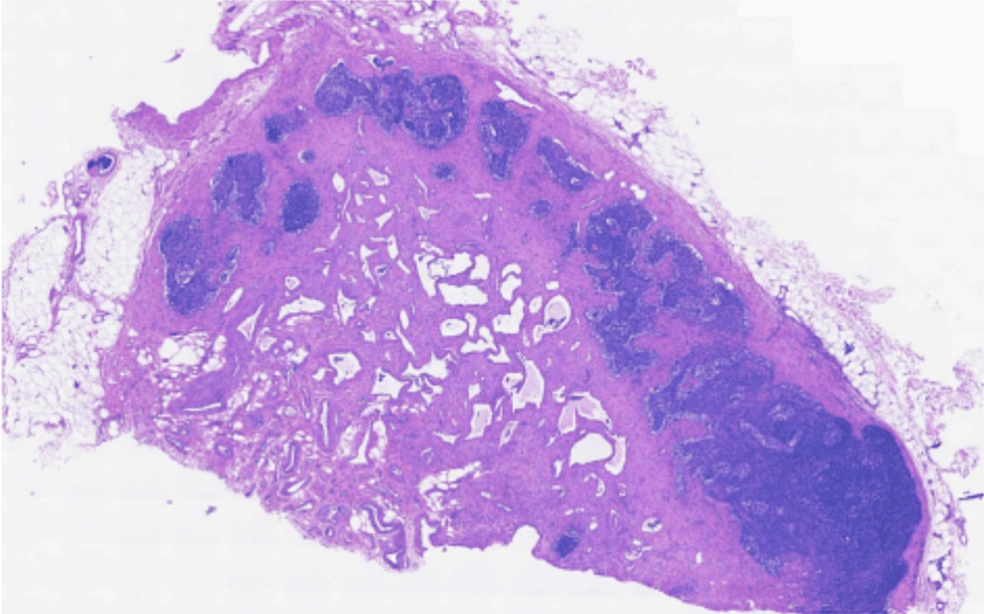
Histopathological section showing no gross lesions and suggestive of intranodal hemangioma

A diagnostic assessment of bilateral lymphadenopathy was initially made with ultrasound, which revealed absent left testicle inguinal soft tissue. It also exhibited multiple enlarged right and left inguinal lymph nodes, with the largest one located on the left and measuring 1.8 x 1.3 cm. There was no presence of a solid or cystic mass, fluid collection, or hernia. Because of these results, the surgeon found it appropriate to consider a left inguinal lymphadenectomy with a frozen section. After a biopsy of the largest left inguinal lymph node, the pathology report was returned as lymphangioma, negative for lymphoproliferative lesions (Figure 1).

The planned intervention included an excisional biopsy of the largest lymph node on the left side. The risks and benefits were explained to the patient, including the risk of infection and bleeding, as well as other alternatives, to which the patient agreed. All preoperative labs and consents were gathered, and the procedure was carried out.

During postoperative day nine of the excisional biopsy of the left inguinal mass, the patient returned to the clinic for a follow-up visit, where he was informed of the biopsy results and notified of his diagnosis of benign lymphangioma. During the visit, the patient denied fever, chills, sweats, unintentional weight loss, fatigue, penile discharge, bleeding, or being malodorous. He also denied any worsening of the swelling of the right inguinal region. There was no discharge, bleeding, or swelling at the surgical incision site upon physical examination. On further examination, there was no penile swelling, and the bilateral inguinal regions were non-tender to palpation. The left inguinal surgical scar was noted to be healing well. No swelling, bleeding, discharge, induration, erythema, or warmth were noted. No skin changes or ulcerations were observed. The patient was advised to come to the emergency department in case of increased swelling or signs of infection. He was further advised to schedule an appointment if any increases in swelling were noticed in the right inguinal region.

## Discussion

Lymphangiomas are benign malformations of the lymphatic system on the skin or mucous membranes. They consist of thin-walled, cystically dilated vascular channels lined by unremarkable endothelial cells and containing proteinaceous lymph fluid. Typically, these clusters of dilated lymphatic channels are found within the superficial dermis and can present as purple or dark red skin lesions in areas with lymphedema [2]. However, they can be found to extend upwards into the epidermis, producing a benign overgrowth of the stratum spinosum [1]. In the United States, lymphangiomas encompass 4% of all vascular tumors and 6% of all pediatric benign tumors (25% of all benign pediatric vascular tumors), and there is no associated increased risk for them based on gender or race [3].

These lymphatic deformities can present due to congenital causes or acquired causes. Congenital causes of lymphangioma development include errors in the lymphatic system's development process, such as abnormal budding of lymphatic vessels and a lack of fusion with the venous system. Other causes of congenital lymphangiomas include detachment or separation of lymphatic tissue and obstruction of lymphatic vessels. Acquired lymphangiomas are usually due to minor defects in the lymphatic system present simultaneously with pathologic conditions, therefore complicating and inhibiting compensation for the defects [1].

Although the definite pathogenesis of lymphangiomas is still unclear, there are suspected mechanisms through which they originate, most involving errors that occur throughout the development of the lymphatic system. Currently, there are three main theories of lymphatic system development: the theory of centrifugal development, the theory of centripetal development, and the theory of combined venous-mesenchymal origin [4,5].

The theory of centrifugal development states that the lymphatic system's origin is the budding embryonic veins of the precursory venous system. This occurs as a result of the sprouting of endothelial cells from veins into neighboring tissue, which leads to the coating of perivascular intracellular gaps with venous endothelial cells. The theory of centripetal development contends that peripheral lymphatic vessels are derived from alleged mesenchymal lymphangioblasts. This is attributed to the fusion of mesenchymal lymphangioblasts, which form the lymphatic vessels, followed by their integration into veins. The theory of combined venous-mesenchymal origin asserts that central lymphatic vessels are of venous origin and are formed by budding from lymphatic sacs, while peripheral vessels have mesenchymal origin and develop by in situ differentiation of mesenchymal progenitors.

Lymphangioma diagnoses are mainly based on patient history and physical exam, but biopsies and dermoscopies can be used as confirmatory tests. Imaging such as MRI and CT can help determine the depth and size of the mass. Around 50% of lymphangiomas are diagnosed at birth, but 90% are diagnosed before the age of two. It is estimated that 75% of lymphangiomas are diagnosed in the head and neck, and about 20% are axillary [6].

Initially, lymphangiomas were categorized based on histological features, giving the subtypes of cavernous, capillary, and cystic. The cavernous and cystic lymphangiomas are considered to be deeper lesions, while lymphangioma circumscriptum and acquired lymphangiomas are more superficial. More recently, though, they have been recategorized based on their morphological traits, giving rise to macrocystic (> 1 cm), microcystic (< 1 cm), and combined subtypes [7].

Clinically, lymphangiomas enlarge slowly and painlessly, but they can have different presentations according to their type and location [5,8]. Lymphangioma circumscriptum presents as clustered or spread-out vesicular papules that can be either translucent or hemorrhagic. These lesions can be found near the genitalia, axilla, and inguinal areas. Unlike the other types of lymphangiomas, these acquired lesions commonly present with lymphedema and can be accompanied by burning pain, pruritus, lymphatic drainage, infection, and aesthetic complications. Cavernous lymphangiomas usually appear during infancy and are poorly defined and painless. Usually, they are not accompanied by changes in the skin layer over them and can be characterized as a subcutaneous bulge but may be tender upon deep palpation. Cystic hygromas have a more defined shape than cavernous lymphangiomas and are most commonly found on the groin, axilla, or neck. Upon palpation, these malformations are soft and vary in both size and shape [1]. Overall, most clinical symptoms are due to mass effects and are primarily dependent on the location of the lesion. For example, when present on the floor of the mouth, oropharynx, and neck, airway complications may be present. Lymphangiomas in the cervical region can lead to compression of the mediastinum, pharynx, and trachea, causing breathing difficulties. When in the laryngeal region, they may cause airway occlusion [1].

Although lymphangiomas most commonly have benign and nonthreatening presentations, lymphangioma circumscriptum can be complicated by cellulitis and lymphatic fluid leakage [3,4]. Even more infrequently, there have been resultant squamous cell carcinomas, verruciform xanthomas, and lymphangiosarcomas that have been associated with lymphangiomas. Additionally, acquired lymphangiomas are at risk for complications due to bacterial infections, as they can have poor lymphatic drainage [1,9,10].

Most lymphangiomas can be subject to observation and conservative treatment unless functional compromise arises, in which case excision would be indicated. Alternatives to surgery include destructive treatments such as sclerotherapy, direct injection with 1% or 3% sodium tetradecyl sulfate, doxycycline, or ethanol, laser therapy, cryotherapy, and superficial radiotherapy [4,6,7].

## Conclusions

As most lymphangiomas are found in the head, neck, and axillary regions, the inguinal lymphangiomas found in this patient make for a very rare presentation. Furthermore, this case is unique as it is the first documented case of an adult who presents with bilateral lymphangiomas in the inguinal region. Lymphadenopathies are clinical findings suggestive of a clinical process in that particular region of the body. Ruling out common causes like inflammatory processes and proliferative disorders is essential to generating a proper treatment plan. This case implicates the need to consider lymphangiomas in a working differential in patients with masses in areas with high lymphatic activity, including the inguinal region, despite age.
